# RBSP‐ECT Combined Spin‐Averaged Electron Flux Data Product

**DOI:** 10.1029/2019JA026733

**Published:** 2019-11-09

**Authors:** A. J. Boyd, G. D. Reeves, H. E. Spence, H. O. Funsten, B. A. Larsen, R. M. Skoug, J. B. Blake, J. F. Fennell, S. G. Claudepierre, D. N. Baker, S. G. Kanekal, A. N. Jaynes

**Affiliations:** ^1^ New Mexico Consortium Los Alamos NM USA; ^2^ Los Alamos National Laboratory Los Alamos NM USA; ^3^ Institute for the Study of Earth, Oceans and Space University of New Hampshire Durham NH USA; ^4^ The Aerospace Corporation El Segundo CA USA; ^5^ Department of Atmospheric and Oceanic Sciences University of California Los Angeles CA USA; ^6^ Laboratory for Atmospheric and Space Sciences University of Colorado Boulder Boulder CO USA; ^7^ NASA Goddard Space Flight Center Greenbelt MD USA; ^8^ Department of Physics and Astronomy University of Iowa Iowa City IA USA

**Keywords:** Van Allen Probes, radiation belts, ECT, MagEIS, REPT, HOPE

## Abstract

We describe a new data product combining the spin‐averaged electron flux measurements from the Radiation Belt Storm Probes (RBSP) Energetic Particle Composition and Thermal Plasma (ECT) suite on the National Aeronautics and Space Administration's Van Allen Probes. We describe the methodology used to combine each of the data sets and produce a consistent set of spectra for September 2013 to the present. Three‐minute‐averaged flux spectra are provided spanning energies from 15 eV up to 20 MeV. This new data product provides additional utility to the ECT data and offers a consistent cross calibrated data set for researchers interested in examining the dynamics of the inner magnetosphere across a wide range of energies.

## Key Points

- A new combined electron flux data product for the Van Allen Probes mission is described
- Results from cross calibration of the RBSP‐ECT instrument suite are presented
- This data product represents the first ever complete electron spectra throughout the inner magnetosphere from tens of electron volts to tens of megaelectron volts

## 1 Introduction

Launched in August 2012, the Van Allen Probes have spent more than 6 years studying the dynamics of the inner magnetosphere. Both spacecraft feature a comprehensive suite of instrumentation to study the particles, waves, and fields in the radiation belts (Mauk et al., 2014). A key part of this instrumentation is the Energetic Particle Composition and Thermal Suite (ECT; Spence et al., 2013), which provides particle measurements of both energetic electrons and ions from ≈15 eV up to 20 MeV. The suite consists of three instruments: the Helium Oxygen Proton Electron mass spectrometer (HOPE; Funsten et al., 2014), the Magnetic Electron Ion Spectrometer (MagEIS; Blake et al., 2014), and the Relativistic Electron Proton Telescope (REPT; Baker et al., 2014).

Currently, the data are provided separately for each of the ECT instruments, with spin‐averaged fluxes (included in the level two files) and pitch angle‐resolved fluxes (included in the Level 3 files) available at the spin time cadence (≈11 s). However, many studies using the ECT data are interested in combining data from multiple ECT instruments, which can introduce additional challenges including cross calibration, time cadence issues and different modes of operation. Over the course of the Van Allen Probes mission, there has been a considerable effort by each of the instrument teams to understand instrument responses and improve the agreements between the instruments of the suite. Building on this work, in this paper we describe our efforts to formalize the cross calibration the ECT instruments and produce a combined, spin‐averaged electron flux data product that spans the entire ECT energy range. This is not meant to replace the currently available data products from each of the instruments, but rather to provide a useful and complementary data set for the entire ECT suite. The combined data product described here will be the first in a series of new ECT data products. Work is underway to continue the methods outlined here to produce both a combined pitch angle‐resolved product and ultimately a phase space density product in adiabatic coordinates.

The remainder of the paper is structured as follows: In section 2, we give a brief overview of the ECT instruments' operation and data availability. In section 3 we describe our techniques for cross calibration of the ECT instruments. In section 4 we provide a description of the fitting routine used in the combined data product. In section 5 we explore potential scientific applications for this data product. In section 6 we describe known caveats and issues with the data product. Finally, in section 7 we provide a summary.

## 2 ECT Instrument Descriptions

The Van Allen Probes mission was launched in August 2012 into a near geosynchronous transfer orbit to study the dynamics of the inner magnetosphere including the radiation belts, ring current, and plasma populations. As part of the mission, the ECT instrument suite have operated continuously since October 2012, providing measurements of the energetic particle environment. Over the early part of the mission, the instruments underwent several major changes to energy channel definitions, operational modes, and flux conversion factors. Therefore, in the subsequent sections, we will focus on data from September 2013 onward, when the major operational changes were mostly complete. A brief discussion of the treatment of the data from the early part of the mission can be found in section 6. Major updates to the ECT data are provided in data “releases.” All of the discussion and figures in this paper are based on the latest release of the ECT data, which at the time of this writing are rel04 for HOPE and MagEIS and rel03 for REPT. In this section, we provide a brief overview of the operation and data available from each of the ECT instruments.

### 2.1 HOPE

The lowest energy instrument of the ECT suite, HOPE measures ∼15‐eV to 51‐keV electrons by utilizing time‐of‐flight mass spectrometry with channel electron multiplier detectors (Funsten et al., 2014). As the instrument name suggests, HOPE measures ions (Helium, Oxygen, and Protons) as well as electrons. The instrument measures electrons and ions on alternating spacecraft spins, thus giving a 22.7‐s cadence for electron measurements. During each electron spin, electrons are measured in 72 energy steps over 11.3 s. HOPE has three different operational modes that affect the energies measured in these 72 steps. The first is apogee mode, which is the normal mode of operation where the instrument measures the full range of energies from 15 eV to 51 keV. The second is perigee mode, where the lowest energies (<25 eV) are not measured. The final is burst support mode, where the instrument measures a small subset of energies at higher time resolution. For the combined ECT data product, in order to have a uniform set of energy channels, only the data from apogee mode is used.

HOPE is composed of five polar look directions which coupled with the spacecraft spin allow for measuring a wide range of pitch angles and azimuthal angles. For the combined data product, omnidirectional fluxes from HOPE rather than the spin‐averaged fluxes are used. The omnidirectional fluxes are obtained by integrating over the pitch and gyroangle distributions. A more complete description of the difference between spin‐averaged and omnidirectional fluxes can be found in the supporting information. Due to HOPE's unique data acquisition method, the omnifluxes have undergone more validation and offer a better comparison to the MagEIS measurements. The difference between the HOPE spin‐averaged and omnidirectional fluxes is typically very small (<20% difference 95% of the time).

The latest release of the HOPE data (rel04) includes a correction to account for long‐term changes in the HOPE detection efficiencies. Using onboard measurements of the absolute detection efficiency (Funsten et al., 2005), this correction normalizes the measurements back to the efficiency in the beginning of the mission (late 2012). This correction is computed daily for ions and electrons and smoothed over a 42‐day period to avoid discontinuities. As described later in section 3, the HOPE team is looking into refinements of this correction, including adding shorter timescale corrections for some events.

### 2.2 MagEIS

The middle energy ECT instrument, MagEIS, utilizes a strong magnet (550–4,800 G) to steer energetic electrons into a set of solid state detectors, providing measurements of ∼30‐keV to 4‐MeV electrons across 21 energy channels (Blake et al., 2014). MagEIS consists of four separate instruments on each spacecraft: one unit to measure low‐energy electrons (LOW; ∼30–200 keV), two units to measure medium‐energy electrons (M75 and M35; ∼200 keV to 1 MeV), and one unit to measure high‐energy electrons (HIGH; ∼1–4 MeV). The LOW, M75, and HIGH units all share the same look direction, 75° from the spacecraft spin axis. The M35 unit, which points 35° from the spin axis provides additional pitch angle coverage for the medium‐energy electrons. For the combined data product (along with all currently available MagEIS data products), only the data from the LOW, M75, and HIGH units are included. Current plans are for future data products, including the combined pitch angle‐resolved data product, to utilize the M35 measurements. While they have remained constant since September 2013, the MagEIS energy pass bands and channel assignments have changed with time. For a complete history of these changes, see the ECT web page (https://rbsp-ect.lanl.gov/science/DataQualityCaveats.php).

As described in Claudepierre et al. (2015, 2019), the MagEIS data have recently undergone an extensive background correction. Wherever possible, the background‐corrected fluxes from MagEIS are used in the combined data product. However, there are various periods where the instrument is operated in high‐rate mode and the background correction cannot be performed. Rather than leave large gaps in the spectrum, during these times, the uncorrected fluxes on MagEIS‐LOW and MagEIS‐M75 are used. Whenever the uncorrected fluxes are used, this is indicated with a data qualify flag. This substitution is made only when the background‐corrected fluxes were not available. In the event that the fluxes were set to 0, indicating that the signal was dominated by background, the uncorrected fluxes are not used.

### 2.3 REPT

The highest‐energy instrument in the ECT suite, REPT, uses a stack of solid state detectors to provide measurements of high‐energy electrons (Baker et al., 2014). Electron measurements are provided in 12‐energy channels ranging from 1.8–20 MeV, with the two highest energy channels acting as integral channels (≥15 MeV and ≥18.9 MeV). REPT was the first of the ECT instruments to turn on and has operated continuously since September 2012. In order to minimize penetrating backgrounds, REPT includes a collimator and thick (>10 mm) multilayered side and rear shielding. Due to this design, throughout the outer zone, REPT generally provides a clean measurement of the high‐energy part of the spectrum. However, when fluxes are low, there is a measured background due at least in part to galactic cosmic rays. In the inner zone, the electron measurement suffers from background contamination due to the high‐energy proton population. Work is underway to understand and correct for these backgrounds in a later data release. For the combined data product, treatment of these backgrounds is described in sections 3.3 and 6. Over the course of the Van Allen Probes mission, significant work by both the MagEIS and REPT instrument teams have greatly improved the cross calibration (e.g., O'Brien et al., 2015) and led directly to the results shown in the next section.

## 3 Methodology

In this section, we describe the cross calibration and data selection techniques that were used to generate the combined data product. As mentioned above, the combined data product has a 3‐min time cadence. Therefore, the first step is to do a 3‐min average on all of the fluxes, taking the 10% trimmed mean of each time bin. Time labels are placed at the beginning of each bin. This time averaging offers three main benefits: (1) provides a common time base for all the instruments, (2) improves the counting statistics, and (3) facilitates analysis of longer time periods.

The cross calibration focus on three areas: (1) the HOPE‐MagEIS cross calibration, (2) MagEIS‐REPT cross calibration, and (3) background correction at the REPT high energies. As described above, each of the ECT instruments utilizes different detection techniques that are appropriate for different energy regimes. Therefore, in order to produce consistent and valid spectra, the basic methodology in each of these areas is the same: (1) remove data with high backgrounds and/or poor counting statistics. (2) Where the instrument energy ranges overlap, select the most appropriate and reliable measurements at each energy range. We begin with a discussion of the HOPE‐MagEIS cross calibration.

### 3.1 HOPE‐MagEIS Cross Calibration

**Figure 1:**
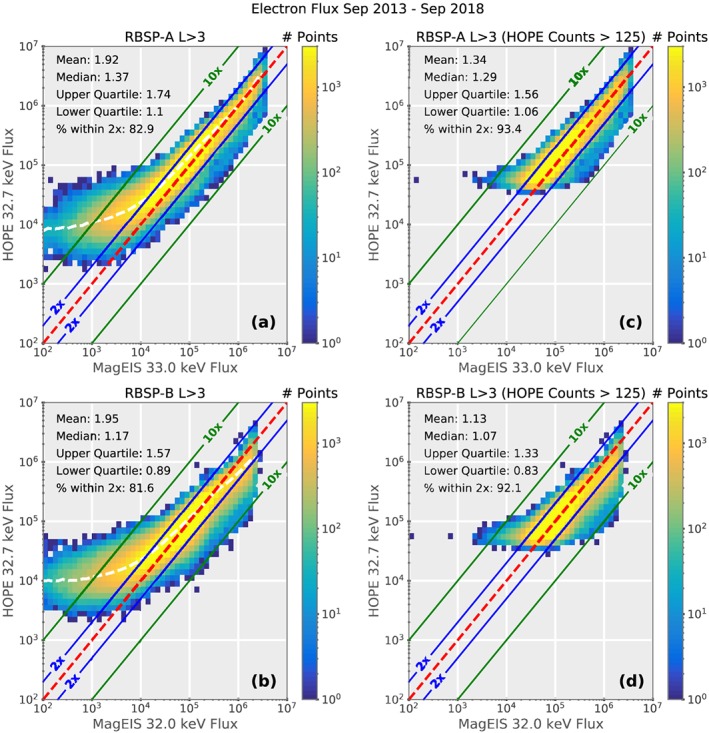
Comparison of the 3‐min‐averaged fluxes from the 31‐/32‐keV channel from MagEIS on RBSP‐A/RBSP‐B with the 32.7‐keV channel from HOPE. The color bar shows the number of measurements in each bin and the colored lines show the boundaries for 1 (red), 2 (blue), and 10 (green) times differences. The white line shows the mean value in each column. Panels (a) and (b) show the comparison with all points included, and panels (c) and (d) show only points where HOPE measured more 125 counts in the 3‐min window. RBSP = Radiation Belt Storm Probes; HOPE = Helium Oxygen Proton Electron mass spectrometer; MagEIS = Magnetic Electron Ion Spectrometer.

MagEIS has two electron energy channels that overlap with the HOPE energy range at 32 and 51 keV. The absolute detection efficiency for electrons on HOPE falls off rapidly for energies greater than 1 keV. As a consequence, the high‐energy channels generally have a low‐detection efficiency and can often suffer from poor counting statistics. Therefore, in the event of disagreement in the overlap region, we will defer to the MagEIS measurements. Figure 1 shows a comparison of the 32‐/33‐keV channel on MagEIS with the 32.7‐keV channel on HOPE. As shown in panels (a) and (b), the agreement between the instruments is very good, particularly at higher fluxes. However, at lower fluxes, the HOPE measurements begin to plateau as the instrument begins to run out of counts, resulting in a poor agreement with MagEIS. In order to avoid these times, we only consider times where HOPE records at least 125 counts in the 3‐min time window, which corresponds to a count rate of three to four counts per second.

Figures 1c and 1d show the comparison after applying this count threshold. For these high count points, there is excellent agreement with MagEIS, with more than 90% of points agreeing within a factor of 2. This same count‐level restriction is applied to all of the HOPE channels with energies greater than 5 keV. Unfortunately, placing the flux cutoff at this level means that only ∼65% of the HOPE electron fluxes at 32 keV and ∼49% at 51 keV are included. While this is a significant reduction in the available data, it is necessary to improve the cross calibration and ensure that the flux spectrum in the overlap region is reliable.

**Figure 2:**
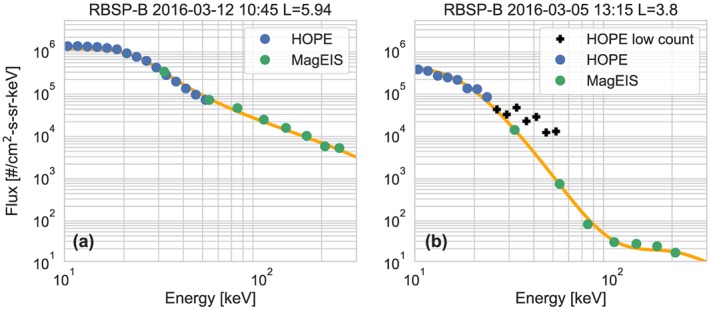
(a and b) Examples of spectra in the HOPE‐MagEIS overlap region. The black crosses show HOPE data that were not included because the counts were too low (<125). The yellow line shows the fit combined flux spectra described in section 4. RBSP = Radiation Belt Storm Probes; HOPE = Helium Oxygen Proton Electron mass spectrometer; MagEIS = Magnetic Electron Ion Spectrometer.

Simply placing the count threshold on HOPE (above 5 keV) results in excellent agreement in the overlap region 96% of the time. Figure 2 shows two examples of these spectra. In particular, Figure 2a shows an example of the excellent agreement when all HOPE energies are included. In Figure 2b, all of the HOPE channels above 25 keV have too few counts (indicated by the black crosses) and differ significantly from the nearby MagEIS channels. Removing these points provides a consistent and reliable spectra in the overlap region.

**Figure 3:**
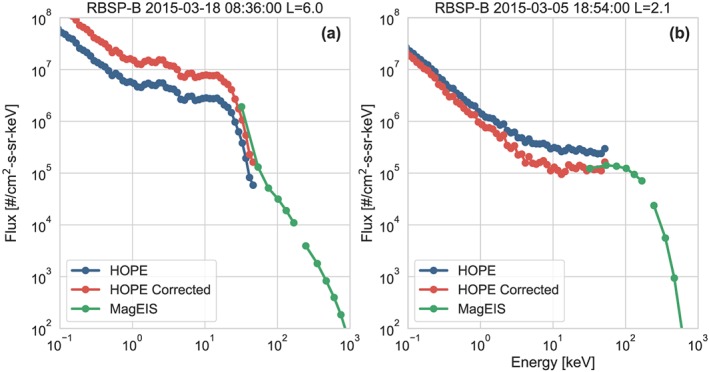
Examples of corrective scaling applied to HOPE. Panel (a) shows the correction for March 2018 where there is a large disagreement between HOPE and MagEIS. All HOPE energies are scaled by the same factor. Panel (b) shows a correction in the inner zone. The background counts are identified using the ratio at the 32‐keV channel. The counts are then subtracted from each energy and the flux is recalculated, creating an energy‐dependent correction. RBSP = Radiation Belt Storm Probes; HOPE = Helium Oxygen Proton Electron mass spectrometer; MagEIS = Magnetic Electron Ion Spectrometer.

For the remaining ∼4% of points, the HOPE data require additional corrections in order to better match the MagEIS data. When needed, these are applied as two separate time‐dependent corrections. The first of these is a correction applied at all L‐shells during times when there is a significant disagreement with MagEIS. This correction is only applied to days where the daily median ratio between MagEIS and REPT at 32 keV is <0.4 or >2.5, indicating that more than half of the measurements for that day differ by more than a factor of 2.5. This correction is very uncommon: Through December 2018, it was only needed for 2 days on RBSP‐A and 15 days on RBSP‐B. For these days, the HOPE data are multiplied by the 2‐day running mean of the MagEIS/HOPE ratio. An example of this corrected spectrum for RBSP‐B on 17–18 March 2015 is shown in Figure 3a.

A complete list of these days where the correction factors have been applied is provided in the supporting information. The majority of these days are associated with large geomagnetic activity, and similar changes are observed with the HOPE ion measurements. Preliminary analysis suggests that these events have rapid time variations in the detection efficiency. Corrections for this effect are currently being worked on and are planned to be included in a future data release. Since these efficiencies affect all energies equally, the entire HOPE energy spectrum is shifted by the corrective factor, as shown in the right panel of Figure 3. While several of the events have small corrections (<2), these corrections result in a better cross calibration and help produce a more reliable combined data product.

The second adjustment is a correction for shield‐penetrating background in the inner zone (*L* < 2.5). Generally, the design of the HOPE instrument was successful in minimizing backgrounds throughout the outer radiation belt. However, in the inner zone, there is a significant background due to the population of high‐energy protons. This background is not influenced by the HOPE Electrostatic Analyzer voltage and will therefore add the same number of counts to each energy channel. Therefore, in order to correct for this, the number of background counts in the 32‐keV channel is determined using the ratio to MagEIS. These counts are then subtracted at each energy and the fluxes are recalculated. This results in a larger correction at the highest energies where there are fewer counts. An example of this correction is shown in Figure 3b. During periods where there are no MagEIS data available, the correction is not performed. In order to provide a comparison to the currently available data, the combined effect of these two scaling factors is given in the HOPE_FACTOR variable. Overall, these changes produce a highly accurate flux spectrum across the entire HOPE‐MagEIS energy range.

### 3.2 MagEIS‐REPT Cross Calibration

**Figure 4:**
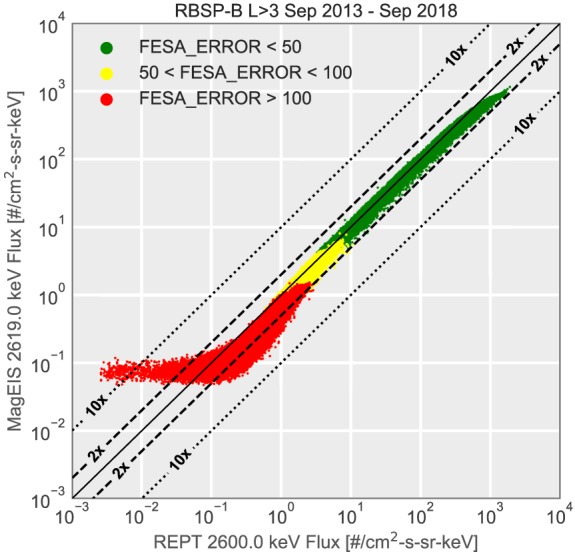
Comparison of the 2.6‐MeV channel on REPT‐B and MagEIS‐B. The points are color coded based on the estimated Poisson counting error in the MagEIS fluxes. The lines show the location of the 1, 2, and 10 times differences. For the combined data product, only fluxes with error <75% (including the green and part of the yellow region) are used. MagEIS = Magnetic Electron Ion Spectrometer; RBSP = Radiation Belt Storm Probes; REPT = Relativistic Electron Proton Telescope; FESA = Flux of Electrons, Spin Averaged.

Next we move to a discussion of the cross calibration of the MagEIS and REPT instruments. There has been considerable effort by both instrument teams to understand the discrepancies between MagEIS and REPT spectral measurements. The first five REPT channels each have a nearby (within 0.2 MeV) MagEIS‐HIGH channel. Similar to HOPE, MagEIS is least sensitive at the highest energies. Therefore, the first step is to identify times where the MagEIS‐HIGH channels have sufficient signal‐to‐noise ratio. Figure 4 shows a comparison of the 2.6‐MeV channels from MagEIS and REPT on RBSP‐B. The color of the points corresponds to the FESA_ERROR variable (“FESA”: Flux of Electrons, Spin Averaged), which gives the estimated error based on the MagEIS counting statistics. For the uncorrected data, FESA_ERROR corresponds to simple Poisson counting statistics, while for the background‐corrected data, there is an additional term related to the background contamination (for a detailed description, see Claudepierre et al., 2015). As shown in Figure 4, when the error is large (red points; indicating a low count rate), the MagEIS fluxes begin to plateau. When the errors are small (shown in green), the measurements from the two instruments generally agree. Therefore, in order to avoid these low count times, the MagEIS‐HIGH fluxes are only used when the estimated error is less than 75%.

**Figure 5:**
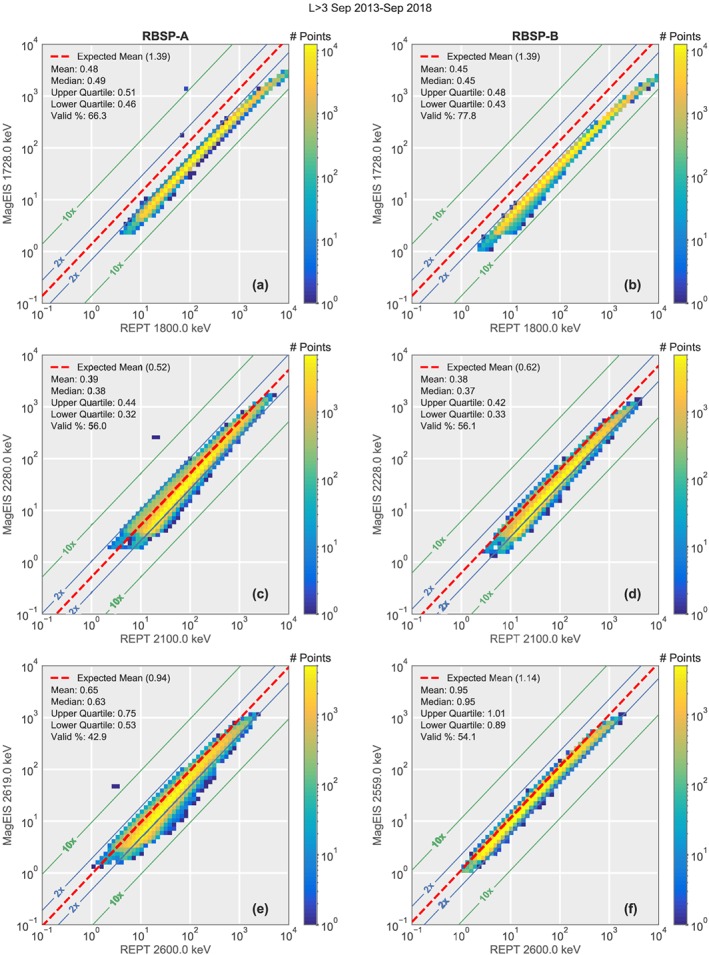
Comparison of the first three energy MagEIS/REPT energy pairs from September 2013 to September 2018. Panels (a), (c), and (e) show data from RBSP‐A, panels (b), (d), and (f) from RBSP‐B. In all the panels the red dashed line indicates the expected mean, given an E^−8^ power law spectrum. The 2, 5, and 10 times lines have been shifted based on this value. In each panel, the mean, median, and quartile values refer to the MagEIS/REPT ratio and the valid % refers to the fraction of the total points where the estimated MagEIS error is less than 75%. MagEIS = Magnetic Electron Ion Spectrometer; RBSP = Radiation Belt Storm Probes; REPT = Relativistic Electron Proton Telescope.

A comparison of the first three energy channel pairs for times that the MagEIS errors are small is shown in Figure 5. The flux spectrum in this energy range (approximately a few MeV) is typically steeply falling as a function of energy. Therefore, some systematic differences in the measured flux between MagEIS and REPT are expected due to the small energy differences (<0.2 MeV) in the channels. In each of the panels, the red dashed line shows where the mean would be expected, given an E^−8^ power law spectrum, which is typical for this energy range (see supporting information). Additionally, we note that the REPT energy channels are obtained using the bowtie approach (Selesnick & Blake, 2000; Van Allen et al., 1974), while the actual channel efficiencies are energy dependent.

Panels (a) and (b) show the first energy pair at  1.8 MeV. For both RBSP‐A and RBSP‐B, the fluxes in REPT channel are consistently higher than those measured in the adjacent MagEIS channel by more than a factor of 2. One potential source for this disagreement is the long tail in the energy‐dependent efficiency of REPT for this channel which can lead to higher measured flux values. For the combined data product, in order to produce a consistent spectrum, this difference needs to be addressed in some way. Therefore, we chose to not include data from the first REPT energy channel (1.6–2.0 MeV) in the combined ECT spectrum and use only the measurements from MagEIS to cover this energy range (1–2 MeV).

Panels (c)–(f) show next two energies ( 2.1 and  2.6 MeV), where the comparison is generally very good, with 90% of fluxes agreeing to within a factor of 2. This is especially true for the 2.6‐MeV channels, where the nominal bin energies of MagEIS and REPT are very close together and the REPT energy‐dependent efficiency is much more peaked and narrow. For the next two energy channel pairs at 3.6 and 4.2 MeV, the fluxes agree similar to what is seen at lower energies. However, at these energies, only a small fraction (<20%) of the MagEIS measurements have sufficient foreground signal to satisfy the error requirements. Therefore, while the MagEIS measurements at these energies give additional confidence to the REPT measurements, the highest two MagEIS channels are not included int the combined ECT spectrum and REPT is used to cover this energy range (>3 MeV).

**Figure 6:**
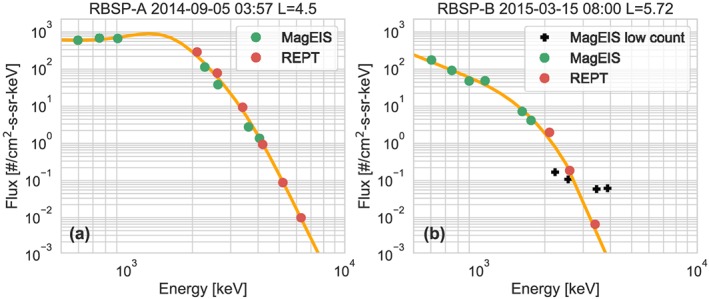
(a and b) Examples of spectra in the MagEIS‐REPT overlap region. The black crosses show MagEIS points that are not included because the Poisson counting error was too large (>75%). The yellow line shows the fit combined flux spectra described in section 4. MagEIS = Magnetic Electron Ion Spectrometer; RBSP = Radiation Belt Storm Probes; REPT = Relativistic Electron Proton Telescope.

Overall, the agreement between MagEIS and REPT is very good and produces a reliable spectrum in the overlap region. Two examples of these spectra in the MagEIS‐REPT overlap region are shown in Figure 6. In particular, the spectrum shows excellent agreement both when all the MagEIS channels are included Figure 6a and when several of the MagEIS channels are not included due to large estimated errors (Figure 6b). In the next section we discuss the final cross‐calibration region: background correction for the high‐energy REPT channels.

### 3.3 REPT Background Correction

Throughout the outer zone, there are times when the REPT energy channels have very low counts and the measurements are dominated by backgrounds that are most likely due to galactic cosmic rays. While the galactic cosmic ray background will affect all energy channels equally, it is especially noticeable at the higher energies (>5 MeV), which are often closer to their noise floor. This results in a flattening of the spectrum, an example of which is shown in the supporting information. In this section, we describe our technique for identifying and removing these times.

The galactic cosmic ray background is not constant and varies slowly over solar cycle timescales (e.g., McDonald, 1998; Zhang et al., 2015). In order to identify and remove the background‐dominant times, we fit the background using the following function:

(1) $$E(t)=A+B\sin\frac{\pi }{C}t+D\cos\frac{\pi }{C}t$$

where *E* is the background fit, *A*, *B*, *C*, and *D* are the fitting coefficients, and *t* is time. In order to minimize the foreground signal, the fit is calculated using only the fluxes near apogee (L>6). The data are fit twice, first using all times, then a second time excluding the data that was greater than 2*E. This second fit is performed to ensure that the background fit is not overestimating and eliminating too much data, which is particularly important for the lower energy channels. After the second fit, all fluxes (across all L‐shells) less than 2*E are marked as invalid. This technique is only used for REPT channels 4–12 (>4.2 MeV). For the lower energy channels, the foreground signal is always much stronger than the background, so no correction is needed. Therefore, for these channels, similar to what was done for MagEIS, the fluxes are marked invalid if they are near the three‐ to four‐count level.

**Figure 7:**
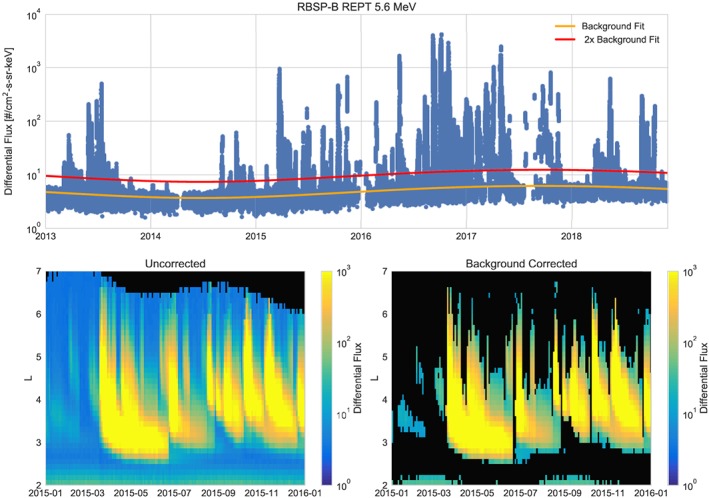
Example of the background fit for the 5.6‐MeV channel on RBSP‐B. The top panel shows the data from near apogee (*L*>6) with the background fit (orange line) and 2 times the background fit shown in red. The bottom left panel shows an L‐sorted spectrogram of the uncorrected data; the bottom right show the data after the fit is applied. Points where the fluxes were below the background fit are shown in black. RBSP = Radiation Belt Storm Probes.

An example of this background removal for the 5.6‐MeV channel is shown in Figure 7. As shown in the L‐sort spectrogram in Figure 7 the fluxes are only affected if they are at or below background levels. At the lower energies (4.2 and 5.6 MeV), this removes ≈30–40% of the data. As would be expected at higher energies, a larger percentage is at or below background levels, including up to ∼90% of the fluxes at or above 9.9 MeV.

## 4 Spectra Fitting

The techniques described in the previous section produces a combined flux spectrum that spans 102 energy channels (72 from HOPE, 19 from MagEIS, and 11 from REPT) across 6 orders of magnitude in energy. In addition to these combined flux spectra, we also provide spectra that have been fit with a spline function. These fit spectra offer several key advantages including providing a consistent set of energy channels for the entire Van Allen Probes mission, eliminating the need to interpolate across changes in the MagEIS energy definitions. In this section, we describe the methodology used to fit the data.

The combined flux spectra are fit in log‐log space using a univariate cubic spline. The fit spectra is output at 127 logarithmically spaced energies between 10 eV and 20 MeV. This equates to 20 energy values per decade, giving comparable energy resolution to the input data in the HOPE energy range, and considerably higher resolution in the MagEIS and REPT energy ranges. The fit is not used to extrapolate; any output energies that fall above or below the upper/lower input energies are set to fill values. The lower‐energy bound is set at the first valid energy, which is typically the lowest energy channel on HOPE. The upper energy bound is L‐shell dependent. In the outer zone (*L* > 2.5), the upper energy limit is set at the last valid energy channel, whereas in the inner zone (*L* < 2.5), the upper limit is set at the last MagEIS‐M75 channel (∼1 MeV; see discussion in section 6 below). Both the input spectra (FESA_INPUT) and the fit spectra (FESA_FIT) are supplied in the data files. All of the fits were generated using the scipy software package (Jones et al., 2001). The knots and coefficients of the spline fit are also provided, allowing users to reproduce the fit and output to any desired energy values using a B‐spine fitting routine. For more details, see the supporting information.

To calculate the fit, two input variables are specified. The first is the weighting of each of the points, which is nominally set to 1.0 for all energy channels with valid fluxes and 0.0 for all invalid fluxes. Additionally, the weighting of some of the energy channels is reduced to 0.5 at all times in order to produce consistent spectra. These include the lowest energy channel of both MagEIS‐M75 and MagEIS‐HIGH, which are often offset compared to the adjacent lower energy channel. Reducing the weight of these channels allows the fit to place more weight on the nearby channels and avoids introducing artifacts into the spectrum. Additionally, the weights for the six highest HOPE energy channels are reduced to 0.5 in order to give preference to the MagEIS channels that generally have better counting statistics.

The second parameter is the smoothing factor *s*. The knots and coefficients of the fit are automatically determined such that the residual of the fit is limited by the following relation:

(2) $$\sum_{i}(w(i)*{\left(y(i)-z(i)\right)}^{2}\leq s$$

where *w* are the weights, *y* is the log of the input fluxes, *z* is the spline fit, and *s* is the smoothing factor. Nominally, for all fits, *s* is set to 0.25. However, each fit undergoes three validation steps where the smoothing can be increased in order to produce a valid spectrum. In all of these cases where the smoothing was increased, the fit spectra should be used with some caution and users are encouraged to consult the data quality flags (FESA_FIT_Quality) to identify these times.

The first of these validation steps is a simple test of the residual of the fit. By definition, as shown in equation (2), the residual should be less than or equal to the smoothing factor. If the residual is greater than 2*s, this indicates that there is a problem with the fit that needs to be corrected. In these cases, the smoothing factor is increased to 0.55. If the residual is still too large, the fit output is set to fill values for all energies. This correction affects less than 0.1% of total fits.

**Figure 8:**
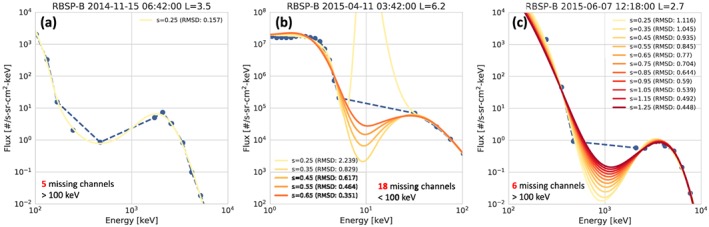
Two examples of fit spectra from RBSP‐B where there is a significant data gap. Panel (a) spectrum with a data gap near 10 keV where the smoothing is increased up to 0.65 to bring the RMSD below 0.4. Panel (b) shows a large dip in the fit spectra due to a data gap in Magnetic Electron Ion Spectrometer M75/HIGH where the smoothing is increased to 1.25 to make the dip more shallow. RBSP = Radiation Belt Storm Probes; RMSD = root‐mean‐square deviation.

The second validation step examines the fit's behavior across gaps in the input spectrum. These intervals are the most common cause for problems, as the fits are not properly constrained by the data and can therefore contain nonphysical features. To identify these times, we look at the number of consecutive missing values in the input spectrum. For times with significant data gaps in the HOPE/MagEIS energy range (>18 channels below 100 keV) or the MagEIS/REPT energy range (>6 channels above 100 keV), the data gap is carried over to the fit spectra and all output energies within the data gap are set to fill. Then, for times with more than four consecutive energy channels are missing (across all energies), the fit is compared to a linear interpolation across the data gap(s) using the root‐mean‐square deviation (RMSD):

(3) $$RMSD=\sqrt{\frac{\sum_{m}{\left(y(i)-z(i)\right)}^{2}}{m}}\leq 0.4$$

where *y* is the interpolation, *z* is the spline fit, and *m* are the missing values. As noted in equation (3), the value of the RMSD must be ≤0.4 in order to be considered valid. Until this condition is met, the smoothing factor is increased by 0.1 up to a maximum of 1.25. If RMSD is still too large, the fit is left as is and indicated with a data quality flag. Overall, ∼5% of the total fits require additional smoothing due to a data gap, and ∼1% of the total fits are cut off with a smoothing of 1.25. Examples of this process are shown in Figure 8. The goal of this correction is not to make the fit match the interpolated spectrum, but rather to use the interpolation to identify times when the fit behaves poorly. Figure 8a shows the most typical example, where no additional smoothing is needed. Figures 8b and 8c show spectra where large data gaps necessitate additional smoothing. In particular, Figure 8b shows a spectrum with a data gap near the HOPE‐MagEIS overlap region. At *s*=0.25, there is a large, nonphysical peak near 10 keV. A small increase of the smoothing to 0.4 largely removes this feature and produces a more reliable spectrum. Finally, Figure 8c shows a spectrum where there is large data gap due to many MagEIS‐M75 and HIGH channels being dominated by background. Therefore, it is reasonable to expect the real spectrum to have a dip at those energies. There is a large increase in the smoothing up to 1.25, which does not remove the dip, but only makes it more shallow, leading to more appropriate flux values at those energies.

The final validation step is to identify and remove times that have nonphysical artifacts such as spikes or step functions. This consists of two checks on the derivatives of the fit. The first is an examination of the fit's second derivative. Typically, the maximum absolute value of the second derivative is ≤75. Therefore, we apply a correction to times when the maximum of the second derivative is twice the typical maximum. For these points, the smoothing is increased by 0.25. If the second derivative is still too large after the increased smoothing then all energies with one decade of the maximum are set to fill values. The second check is on the first derivative at high energies. Any points above 5 MeV that have a positive slope are set to fill values.

**Figure 9:**
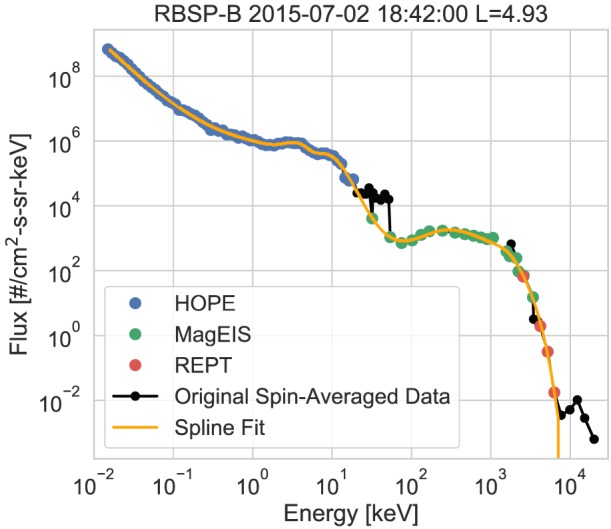
Example of a fit spectrum on RBSP‐B. The blue, green, and red points are the input spectrum, using the methods described in section 3. The orange line is the spline fit of the spectrum and the black points are a simple 3‐min time average of the currently available Energetic Particle Composition and Thermal Plasma data. HOPE = Helium Oxygen Proton Electron mass spectrometer; MagEIS = Magnetic Electron Ion Spectrometer; RBSP = Radiation Belt Storm Probes; REPT = Relativistic Electron Proton Telescope.

An example of a fit spectrum is shown in Figure 9. As shown in the figure, the fit produces a complete smoothed version of the observed spectra. This figure also illustrates the advantages of this data product over a simple combination of the currently available instrument specific spin‐averaged electron fluxes (shown in black). This is particularly evident near the instrument boundaries, where without applying the selection methods in section 3 large discrepancies can exist leading to inaccurate spectral shapes and features. Therefore, we strongly encourage any users interested in utilizing data from multiple ECT instruments to use this data product.

## 5 Scientific Applications

The combined data product described here will greatly benefit scientific studies of the inner magnetosphere and radiation belts. The use of multisatellite observations has been key to many recent studies. The fit spectra in this data set directly facilitate those types of studies, providing a continuous function that can be sampled at different energies for direct comparison to other complementary data sets, including GPS (Morley et al., 2017), LANL‐GEO (Bame et al., 1993; Belian et al., 1992; Meier et al., 1996), THEMIS (Angelopoulos, 2008), and ARASE (Miyoshi et al., 2017). In addition, this data product will provide a compact and consistent data set for use in long‐term statistical studies of the Van Allen Probes era.

One particular type of study that will benefit from this new data product is examination of spectral shapes and features. Recent work by Zhao, Johnston, et al. (2019) and Zhao, Ni, et al. (2019) has identified frequent ‘bump‐on‐tail’ distributions, where there is a local peak in flux at high energy (few megaelectron volts). This peak often falls at or very near the MagEIS/REPT overlap region (1–2 MeV), so combined measurements from both instruments are needed. Examining the fit spectra during these intervals can more accurately and precisely determine where these features are located and how they evolve.

## 6 Known Caveats

In this section, we describe some of the major known caveats of this data product at the time of initial release. For the most up‐to‐date list of known caveats as well as changes in future data releases, users should consult the accompanying documentation available on the RBSP‐ECT website (http://www.rbsp‐ect.lanl.gov/).

### 6.1 Inner Zone

In the inner zone (*L* < 2.5), all the energy channels >1 MeV are not included in the fit and therefore set to fill values. This does not necessarily mean that there are no electrons at those energies at any times. This cutoff was chosen based on the results of Fennell et al. (2015) and Li et al. (2015), which both showed that during the Van Allen Probes era there were no observations of >900‐keV electrons. More recent results from Claudepierre et al. (2019) have shown that >1‐MeV electrons have been observed in the inner zone during limited intervals. However, those results employed a labor intensive background correction that cannot be practically be applied at all times, and the combined data product will continue to utilize a cutoff at 1 MeV.

### 6.2 Measurement Gaps

Throughout the mission there are various times when the measurement from one or more of the ECT instruments is not available. As noted above, during these times the smoothing is often increased to prevent the fit from behaving poorly. However, during these times it is possible that the fit does not accurately reflect the shape of the spectrum across the data gaps and therefore should be used with caution. These intervals can be identified by looking at the FESA_FIT_Quality flag.

### 6.3 Early Mission

The results shown in the above sections, and the initial release of this data product are only for the period of September 2013 onward, when the instrument energy channels and operational modes settled to their final values. Prior to this period, there are various intervals where the energy channels or flux conversion factors change. Data files will also be available back to at least January 2013. For these pre‐September 2013 files, it is likely that there will be some issues with data reliability and several modifications to the methodology used during this period. Users should consult the accompanying documentation available on the RBSP‐ECT website (http://www.rbsp-ect.lanl.gov/).

## 7 Summary

This paper describes a new data product combining the spin‐averaged electron fluxes from all of the ECT instruments. While the currently available ECT data have been used in numerous studies, there are several challenges for studies that utilize data from multiple instruments. The goal was to produce a consistent, reliable and cross‐calibrated data set, representing the first complete electron spectra throughout the inner magnetosphere from electron volts up to tens of megaelectron volts. The combined data set will bring additional utility to the ECT data and will be especially useful for researchers interested in studying effects, which span two or more of the ECT instrument's energy ranges. As new data releases become available, the combined data product will be updated and refined. Any updates to the techniques described here will be available on the ECT web page.

The methodology and techniques described here will also be used to generate higher level combined data ECT products. We are currently working on a combined pitch angle‐resolved flux data product, which will incorporate many of the techniques outlined here. This will eventually lead to an official release of phase space density calculated using the combined ECT data set.

## Supporting information



Supporting Information S1
