# Supplementary material for: RBSP‐ECT Combined Spin‐Averaged Electron Flux Data Product
Source: J Geophys Res Space Phys. 2019 Nov 9;124(11):9124–36. doi: 10.1029/2019JA026733 (PMC6988469; doi:10.1029/2019JA026733)
Supplement: Supplementary file 1 — Supporting Information S1 [file JGRA-124-9124-s001.pdf]

AGU PUBLICATIONS

Journal of Geophysical Research: Space Physics

Supporting Information for

RBSP-ECT Combined Spin Averaged Electron Flux Data Product

A.J. Boyd[1], G.D. Reeves[1,2], H.E. Spence[3], H.O. Funsten[2], B.A. Larsen[1,2], R.M. Skoug[2], J.B. Blake[4], J.F. Fennell[4], S.G. Claudepierre[4,5], D.N. Baker[6], S.G. Kanekal[7], A.N. Jaynes[8]

[1]New Mexico Consortium, Los Alamos, NM, USA, [2]Los Alamos National Laboratory, Los Alamos, NM, USA, [3]Institute for the Study of Earth, Oceans and Space, University of New Hampshire, Durham, NH, USA, [4]The Aerospace Corporation, El Segundo, CA, USA, [5]Department of Atmospheric and Oceanic Sciences, University of California Los Angeles, Los Angeles, CA, USA, [6]Laboratory for Atmospheric and Space Sciences, University of Colorado, Boulder, CO, USA, [7]NASA Goddard Space Flight Center, Greenbelt, MD, USA, [8]Department of Physics and Astronomy, University of Iowa, Iowa City, IA, USA

Contents of this file

Text S1 to S3

Figures S1 to S3

Tables S1 to S2

Introduction

Text S1 and Figure S1 describe the derivation of the E^{-8} power law that was used in the MagEIS-REPT cross-calibration section of the main paper. Text S2 describes the difference between spin-averaged and omni-directional fluxes. Text S3 describes the B-spline basis and some basic information/caveats for reconstructing the provided fit spectra. Figure S2 shows an example of HOPE corrections during the March 2015 storm. Figure S3 shows an example of a flattening spectrum that can occur in the REPT energy range. Table S1 provides a list of the days where additional corrections have been applied to the HOPE data due to a large difference between HOPE and MagEIS. Table S2 lists the variables that can found in each of the data files along with their descriptions.

Text S1. MagEIS/REPT spectral slopes

In the discussion of the MagEIS-REPT cross-calibration in the main text (and in particular Figure 5), we used an E^{-8} power law to compare adjacent MagEIS and REPT channels. As noted in the text, E^{-8} is a typical spectrum at these energies. Here, we describe the process for determining

that value. This was not meant to be a comprehensive study of spectral shapes, but merely to better understand the effect that energy differences have on the measured fluxes.

To determine the typical spectral slope in the overlap region, data from 2 MagEIS channels (~1.7 and ~2.2 MeV) and 4 REPT channels (2.1 MeV, 2.6 MeV, 3.4 MeV and 4.2 MeV) were used. Selecting times above L=4, we fit the spectra using a power law function by taking a linear fit of the log of the fluxes. A histogram of the resulting slopes is shown in Figure S1. While there is a relatively large spread, we chose to use E^{-8} for the comparisons since it corresponded to the median slope observed by both spacecraft.

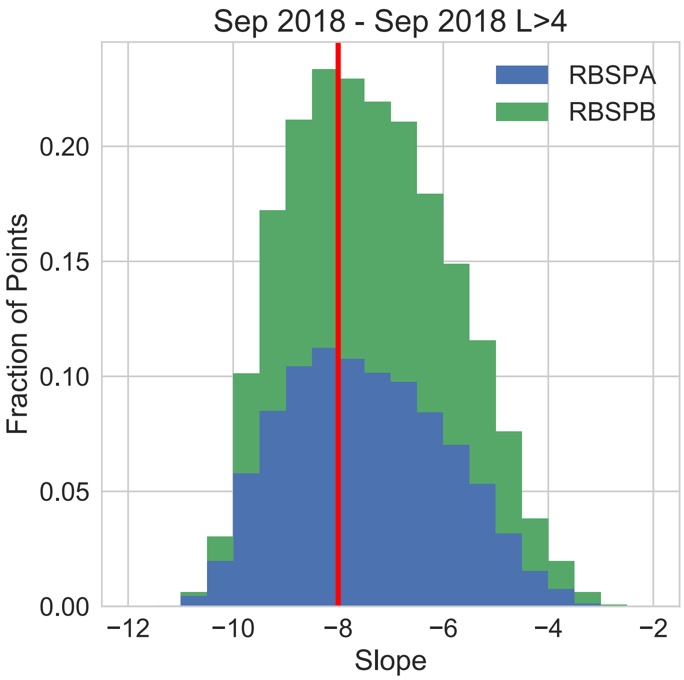

Figure S1. Histogram of the spectral slope determined using a power law fit from 1.7-4.2 MeV. The red line shows the value (-8) that was used in the main text.

Text S2. Omni/Spin-Averaged Fluxes

As noted in Section 2, the omni-directional fluxes are used for HOPE rather than the spin-averaged fluxes. Here, we describe the difference between the two measurements and give further explanation about why the omni-directional fluxes are used.

As the name suggests, the spin-averaged fluxes represent the average flux measured during a across a single measurement period, which is nominally the spin period (~11 seconds). For HOPE, the fluxes from each polar pixel is averaged together, weighted by the nominal fraction of the unit sphere measured by each pixel (18/36/72/36/18 %).

The omni-directional fluxes are derived from the pitch angle resolved data. Each of the HOPE measurements are binned by pitch and gyro angle. The omni-directional flux is generated by

integrating over each the pitch and gyro angle bins. Omni-directional fluxes are not routinely produced for MagEIS and REPT.

Generally, the difference between these quantities is often very small (<20% difference 95% of the time). However, as noted in section 2.1, the HOPE measurements are not directly tied to spacecraft spin period (as they are for MagEIS/REPT), so the spin-averaged product can include an over-sampling of a fraction of the unit sphere. As a result, there is more confidence in the omni-directional fluxes and they should offer a better comparison to the spin-averaged fluxes from MagEIS.

Text S3. B-splines

As mentioned in the main text, the data files contain all of the variables necessary to reproduce the spline fit. The spline S(x) can be defined using a B-spline basis:

$$\boldsymbol{S(x)} = \sum_{j=0}^{n-1} \boldsymbol{c_j B_{j,k;t}(x)}$$

Where t are the knots, c are the coefficients, k is order of the spline (in this case k=3) and B are B-spline basis functions, defined as follows:

$$\boldsymbol{B_{j,0}} = \begin{cases} \mathbf{1}, & \text{if } \boldsymbol{t_j \le x < t_{j+1}} \\ \mathbf{0}, & \text{otherwise} \end{cases}$$

$$\boldsymbol{B_{j,k}} = \frac{\boldsymbol{x - t_j}}{\boldsymbol{t_{j+k} - t_j}} \boldsymbol{B_{j,k+1}} + \frac{\boldsymbol{t_{j+k+1} - x}}{\boldsymbol{t_{j+k+1} - t_{j+1}}} \boldsymbol{B_{j+1,k-1}}$$

The original fits were done using python, but most major programming languages (including C and Matlab) have B-spline routines available that can reconstruct the fit using the knots and coefficients. Both the knots (FESA_FIT_Knots) and coefficients (FESA_FIT_Coeffs) are provided in the data files. The number of knots/coefficients used in each fit varies. However, a fixed number of values are provided at each time step (50), padded values that are set to fill (-1.0e31) should be excluded.

If users are only interested in outputting different energies, we recommend they first explore using a simple interpolation on the provided fit fluxes/energies. However, if they wish to reproducing the fit there are a few key caveats:

1. The fit was produced in log/log space, so the output will be log of flux.
2. The reproduced fit will not include any data gaps which have been added due to gaps in the input data or unphysical features.
3. The reproduced fit may extrapolate slightly beyond the input energies. Outputs at these energies will not necessarily be accurate.

Figure S2. Illustration of the correction of the HOPE-B data for 17-18 March 2015. The left panel shows the MagEIS/HOPE ratio at 32 keV as a function of time for this interval. On 17-18 March, the median ratio goes below 0.4 and the HOPE fluxes are shifted up by the green numbers to compensate (shown with the green points). The right panel shows a spectrum from this interval, where the corrected HOPE spectrum (green) shows considerably better agreement with MagEIS than the uncorrected spectrum (blue).

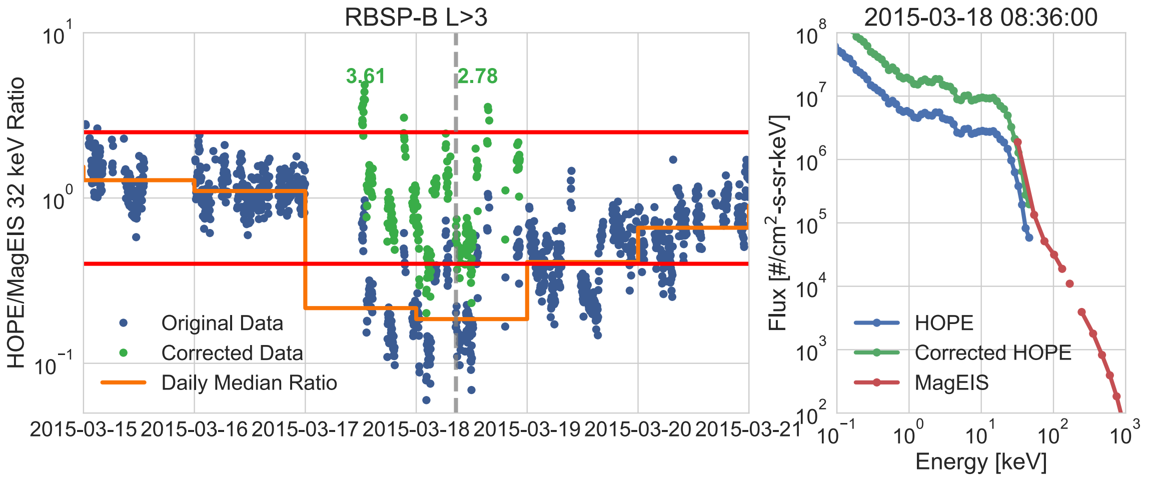

Figure S3. Typical example of REPT flux spectrum, showing the flattening above 5 MeV. The red region shows the part of the spectrum that is removed after the background fit is applied.

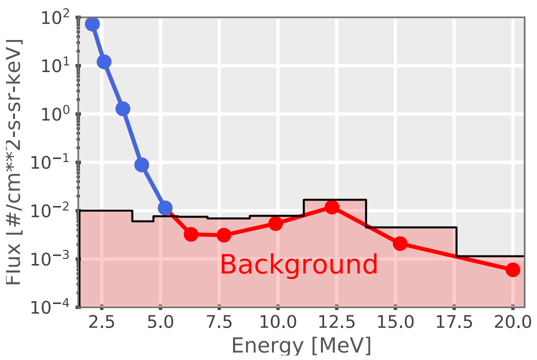

Table S1. List of days from September 2013 – December 2018 where additional corrections were applied to the HOPE data.

Date	Spacecraft	Correction Factor
17 March 2015	RBSP-B	3.61
18 March 2015	RBSP-B	2.78
16 April 2015	RBSP-B	2.07
13 May 2015	RBSP-B	2.36
8 May 2016	RBSP-B	2.16
27 September 2016	RBSP-B	2.07
28 September 2016	RBSP-B	2.21
29 September 2016	RBSP-B	1.83
13 October 2016	RBSP-B	1.93
22 October 2016	RBSP-B	0.36
25 October 2016	RBSP-B	2.04
27 October 2016	RBSP-B	1.78
13 November 2016	RBSP-B	1.92
9 March 2017	RBSP-A	0.38
6 May 2017	RBSP-A	0.29
26 August 2018	RBSP-B	2.58
4 November 2018	RBSP-B	2.15

Table S2. Variable names and descriptions contained in each data file. Some support/metadata variables are not shown here.

Variable Name	Description
B_Calc	Calculated magnetic field strength at spacecraft
B_Eq	Calculated magnetic field strength at magnetic equator
Epoch	Default Time
FESA_FIT	Spline fit combined spin-averaged flux spectra. In the inner zone (L < 2.5), all energies above 1 MeV are set to fill values.
FESA_FIT_Coeffs	Coefficients of the spline fit. Combined with FESA_FIT_Knots, can be used to reconstruct the spline in the B-Spline Basis. If less than 50 coefficients are used the remaining values are set to fill.
FESA_FIT_Energy	Energy values for FESA_FIT
FESA_FIT_Knots	Knots of the spline fit. Combined with FESA_FIT_Coeff, can be used to reconstruct the spline in the B-Spline Basis. If less than 50 knots are used the remaining values are set to fill.
FESA_FIT_Quality	Quality flag for FESA_FIT: 0) Nominal Fit, 1) Increased smoothing due to large residual, 2) Increased smoothing due to data gap, 3) Maximum smoothing due to data gap, 4) Increased smoothing due to large 2nd invariant
FESA_INPUT	Combined spin-averaged flux spectra
FESA_INPUT_Energy	Energy values for FESA_INPUT
FESA_INPUT_Instrument	Flag indicating the instrument corresponding to each FESA_INPUT energy value: 0) HOPE, 1) MagEIS, 2) REPT
FESA_INPUT_Quality	Quality flag for FESA_INPUT: 0) Nominal Data, 1) Uncorrected Data, 2) Data was used in the spline fit at reduced weighting
HOPE_FACTOR	Multiplying factor applied to the HOPE data at all energies. This factor is the combination of two corrections: 1) Inner zone (L < 2.5) 2) Specific days where there is large difference between HOPE and MagEIS.
I	2nd Adiabatic invariant (bounce)
L	Calculated McIlwains L parameter
L_star	Calculated Roederers L* parameter
MLT	Magnetic Local Time
Position	Position of the satellite in geographic coordinates
